# Numbers in Action

**DOI:** 10.3389/fnhum.2016.00388

**Published:** 2016-07-29

**Authors:** Rosa Rugani, Luisa Sartori

**Affiliations:** Department of General Psychology, University of PadovaPadova, Italy

**Keywords:** number, mental number line, SNARC effect, kinematic, action planning, action execution

## Abstract

Humans show a remarkable tendency to describe and think of numbers as being placed on a mental number line (MNL), with smaller numbers located on the left and larger ones on the right. Faster responses to small numbers are indeed performed on the left side of space, while responses to large numbers are facilitated on the right side of space (spatial-numerical association of response codes, SNARC effect). This phenomenon is considered the experimental demonstration of the MNL and has been extensively replicated throughout a variety of paradigms. Nevertheless, the majority of previous literature has mainly investigated this effect by means of response times and accuracy, whereas studies considering more subtle and automatic measures such as kinematic parameters are rare (e.g., in a reaching-to-grasp movement, the grip aperture is enlarged in responding to larger numbers than in responding to small numbers). In this brief review we suggest that numerical magnitude can also affect the *what* and *how* of action execution (i.e., temporal and spatial components of movement). This evidence could have large implications in the strongly debated issue concerning the effect of experience and culture on the orientation of MNL.

In the 19th century Galton first noted that humans visualize and think of numbers as represented on a mental number line (MNL), usually oriented from left-to-right. Along the MNL smaller numbers are located on the left side and larger ones on the right side (Galton, 1880; Dehaene, 2011). The first, and often replicated, experimental demonstration of the MNL is the fact that adult humans are faster at processing small numbers when responses are executed on the left side of space, and at processing large numbers when responses are executed on the right side of space (spatial-numerical association of response codes, SNARC effect; Dehaene et al., 1993). Since this first evidence, large body of studies have investigated the cognitive representation of numbers using chronometric methods, but the impact of number processing on motor control has been scarcely explored. According to a recent influential hypothesis, cognitive representations of perceptual and semantic information cannot be fully understood without considering their impact on actions (Gallese and Lakoff, 2005). In this vein, here we present a critical review to highlight how the existing knowledge on MNL could be fostered by studies that critically analyze the link between motor actions and numbers.

One of the more debated aspects related to the MNL is the origin of its orientation. Because the left-to-right orientation is reduced or even reversed in cultures that read from right to left, it has been suggested that the MNL originates from culturally specific experiences (Zebian, 2005; Shaki and Fischer, 2008; Shaki et al., 2009). Even if the effect of culture in shaping the MNL is undeniable, recently an increasing number of experiments have demonstrated that non-verbal subjects (preverbal infants: de Hevia and Spelke, 2010; de Hevia et al., 2014; Bulf et al., 2016; birds: Rugani et al., 2007, 2010a, 2011, 2014; chimpanzees: Adachi, 2014; and monkeys: Drucker and Brannon, 2014) associate number with space, indicating that the orientation of the MNL could originate from pre-linguistic and biologically determined precursors. In this context a new research highlighted that 3-day-old domestic chicks (*Gallus gallus*), initially trained to respond to a target numerical value (e.g., an array of 5 squares), associated a numerical value smaller than the target (e.g., 2) with the left side of space and a number larger than the target (e.g., 8) with the right side of space, showing a context-dependent spatial-numerical association (Rugani et al., 2015a). These biases emerged also when possible non-numerical cues were controlled (see also, Rugani et al., 2015b, 2016a,b). Interestingly enough, numerical magnitude influenced what responses were selected by chicks, suggesting that the coded magnitude information may reflect a link between numerical processing and actions.

The idea that a connection between numerical processing and action planning does exist arose from evidence that found an activation of the intraparietal sulcus during numerical judgments, motor responses and encoding of spatial information required for motor actions (see Walsh, 2003; Dehaene et al., 2004; Göbel and Rushworth, 2004; Rossetti et al., 2004; Culham and Valyear, 2006; Piazza and Eger, 2016). All these aspects have been integrated in the A Theory Of Magnitude (ATOM) theory (Walsh, 2003). Such a theory suggests that space, number and time would be considered as part of a more general magnitude system, with shared neuroanatomical basis located in neurons of the inferior parietal cortex. In Walsh’s opinion, the efficient organization of spatial, numerical and temporal information within the inferior parietal cortex reflects the need for prompt sensorimotor transformations (Walsh, 2003). In theorizing this, Walsh (2003) was inspired by one of Cajal’s (1898/1999) advise “All natural arrangements, however capricious they may seem, have a function”.

The scope of this functional organization is quite evident when considering it from a comparative point of view. Animals living in natural and wild environments may improve their fitness by rapidly acting; let us think for example at the fight-or-flight responses, that needs to promptly integrate spatial, temporal and also numerical information. A predator would be advantaged to direct its attack toward the larger groups of preys on the one hand, on the other hand a prey would reduce the possibility to be cathead joining the larger group of social companions (Miletto Petrazzini et al., 2012). The latter behavior is so essential that it can be found in domestic chicks since their first days of life (Rugani et al., 2010b). The fact that even newborn birds show context-dependent spatial-numerical association (see also Drucker and Brannon, 2015) suggests that a dedicated network for coding spatial and numerical information develops very early in life following very limited experience. Moreover, this comparative study may inspire the development of new paradigms to study the SNARC from a different perspective. To date, in fact, a large body of literature has replicated the evidence that number processing can modulate response times (which are faster in responding to relatively small numbers on the left and to relatively large numbers on the right), but studies investigating whether number processing affects the selection of the responses are sporadic.

The adoption of a “free response” task could allow to investigate *what* responses are selected, different from previous literature on the SNARC effect, based on forced-choice response paradigms that only provide information about *when* the responses are executed. The first attempt to study this facet of the SNARC was conducted by Daar and Pratt (2008), using a free-response task. They instructed participants to press either a left (z) or a right button (/) on a keyboard as soon as a centrally presented stimulus (that could be a small number: 1 or 2; a large number: 8 or 9; or a neutral character: @, &, *, #) turned from white to green. Therefore, participants were instructed to press whichever button they preferred. Participants produced more left-key presses in responding to small numbers and more right-key presses in responding to large numbers, demonstrating that numerical magnitude affects not only the speed of a response, but also the frequency and direction of the choice. In the same year, Fischer and Campens (2008) investigated the metrical structure of the MNL, by asking adult humans to show with their hands the spatial location of specific numbers. At the beginning of each trial, participants were verbally required to indicate the position of a target number (e.g., “where is number 3?”) and responses were given by pointing with either the left or right index finger to a position corresponding to the target. The location of the pointing was measured with an electromagnetic tracking system. The majority of participants produced a left-right arrangement, associating small numbers with left and larger ones with right space, showing that the spatial numerical association can be detected with a spatial/motor task. Recently, a mutual influence between numbers and motor actions has been found also in real-life contexts (Shaki and Fischer, 2014). In a first experiment, adult humans were required to generate random numbers while walking; on average, they generated more frequently smaller numbers when preparing to turn left. In a second experiment, they were required to stand still and listen to a sequence of numbers, and then to make an immediate turn and walk to either side. Participants turned left more often when they had just listened to small numbers (Shaki and Fischer, 2014).

In these studies, motor responses were induced and recorded to overcome limitations of previous studies based on chronometric responses. However, findings stemming from these innovative paradigms could also be explained by a highly overlearned (and thus very efficient) motor association between numbers and responses (Schwarz and Keus, 2004). In everyday life, in fact, we often perceive and act on spatially-organized numbers, such as rulers and keyboards. The influence of context on the direction of the MNL is indeed a well-known effect: the SNARC can be inverted into a right-to-left direction if numbers have to be imagined on a clock face (Bächtold et al., 1998; see also Vuilleumier et al., 2004). Nevertheless, the evidence that spatial representation of numerical magnitude plays a role in determining which response is selected (Daar and Pratt, 2008; Fischer and Campens, 2008; Shaki and Fischer, 2014) for action is very innovative.

From this fascinating perspective, an essential improvement in the actual literature would be obtained by combining a “free response” task with a kinematic analysis of movement, which may allow to better understand and describe *how* the responses are executed. Kinematic indeed analyses movements in terms of position, displacements (linear and angular), acceleration and velocities of body or segments of body (for a review see Castiello, 2005). Some studies have highlighted that semantic information affects movements: in reaching-to-grasp one of two identical objects, grip aperture was larger when the object was labeled with the word “large” than when it was labeled with the word “small” (Gentilucci et al., 2000; but see also Glover and Dixon, 2002; Glover et al., 2004). With respect to numerical cognition, it has been suggested that hand movements could help children in learning how to solve symbolic additions (Novack et al., 2014; but see Fischer et al., 2015 for a critical comment). From this point of view, studies investigating the relationship between numbers and action (in particular finger movements, see Rusconi et al., 2005) will help to design new paradigms and training to improve numerical comprehension.

To date, only one study has investigated the functional connection between numerical cognition and action planning (Lindemann et al., 2007). In this experiment, participants were required to indicate whether an Arabic digit referred to as odd or even number by means of two different reach-to-grasp movements toward a target object. This object consisted of two segments: the bottom segment was a large cylinder (diameter of 6 cm) and the top segment, glued on the center of the bottom one, was a small cylinder (diameter 0.7 cm). The large segment required a whole-hand power grip to be grasped; the small segment required a precision grip between the thumb and index finger. The target object was placed at the right side of the table behind an opaque screen, allowing participants to reach it easily with their right hand but without visual feedback (see Land, 2006). The required grasping response was counterbalanced between participants: half of participants were required to perform a precision grip in response to even digits (i.e., 2, 8) and a power grip in response to odd digits (i.e., 1, 9), and vice versa for the other half of participants. Whenever the digit 5 was presented, participants were required to refrain from responding. This no-go condition was introduced to ensure that reaching movements were not initiated before the number was processed and the parity judgment was evaluated.

Precision grip movements were initiated faster when responding to small numbers and power grip were initiated faster when responding to large numbers. Moreover, numerical magnitude had an impact on grip aperture kinematics: an enlarged maximum grip aperture was found for power grips associated to larger numbers than for power grips associated to small numbers. Overall, these findings suggest that representation of numbers and action share common codes within a generalized system for magnitude representation (Lindemann et al., 2007). These findings, however, could also be affected by highly-frequent motor associations between magnitude labels (e.g., small, medium, large) and manual responses (e.g., grasping a small or large glass of coke, a 0.5 kg or a 1 kg flour packet).

To sum up, previous everyday experience with rulers and keyboards could prompt to respond to small numbers with the left hand and to larger numbers with the right one. Similarly, frequent experience with numerical labels could explain why we perform smaller grasps in relation to smaller numbers. A first challenge for future studies on SNARC would be therefore to disentangle the role of numerical magnitude from the role of experience. A first step in this direction would be to investigate the relation between number cognition and motor action in a new and not-overlearned task, in which the same behavior has to be performed in response to small, medium or large numbers. Participants could be required to respond to numerical magnitudes, for example by spontaneously directing an object toward one of two identical, but spatially displaced, positions or by directing the object toward the same central position while potential trajectory deviations from the straight line are measured. Accurately measuring performance and kinematic parameters in these tasks would allow to test whether and how numerical magnitude affect action execution. A second step would be to analyze the kinematics of movement that provides an implicit measure of the association between numbers and actions. Kinematics, indeed, would allow to disentangle, from a very innovative perspective, the relative role of experience and culture in shaping the direction of MNL. Moreover, integrating the free-response task with a kinematic analysis of movement would allow to disentangle whether numbers can affect *what* action is selected and *how* the responses are executed.

## Author Contributions

RR and LS developed the concept and wrote the manuscript. Both authors contributed equally to this work.

## Conflict of Interest Statement

The authors declare that the research was conducted in the absence of any commercial or financial relationships that could be construed as a potential conflict of interest.
